# Analysis of Apparent and True Kinetics in Batch Circulation Reactor Systems

**DOI:** 10.1021/acsomega.6c05077

**Published:** 2026-09-10

**Authors:** Reinaldo Giudici

**Affiliations:** Escola Politécnica, Department of Chemical Engineering, 28133 Universidade de São Paulo, Av. Prof. Luciano Gualberto, 380, Travessa do Politécnico 05508-010, São Paulo, SP, Brasil

## Abstract

Kinetic parameter determination in tubular reactors coupled with recirculation tanks is often complicated by kinetic disguises arising from simplified data treatment. When the reaction occurs solely in the tubular reactor but concentration measurements are taken in the stirred tank, treating the system as a batch reactor leads to falsified apparent kinetic parameters. Here we analyze this configuration using asymptotic methods for zeroth-, first-, second- and n*th*- order kinetics, deriving simple equations that relate apparent kinetic parameters to their true values. These relationships enable correction of falsified parameters under defined conditions, revealing that at low conversion per pass, reaction order remains accurate while rate constants are underestimated, whereas at high conversion per pass, the apparent kinetics shifts to first-order with rate constants governed by tank dynamics. This work provides practical tools for accurate kinetic analysis in batch recirculation reactors, improving reactor design and kinetic modeling.

## Graphical abstract

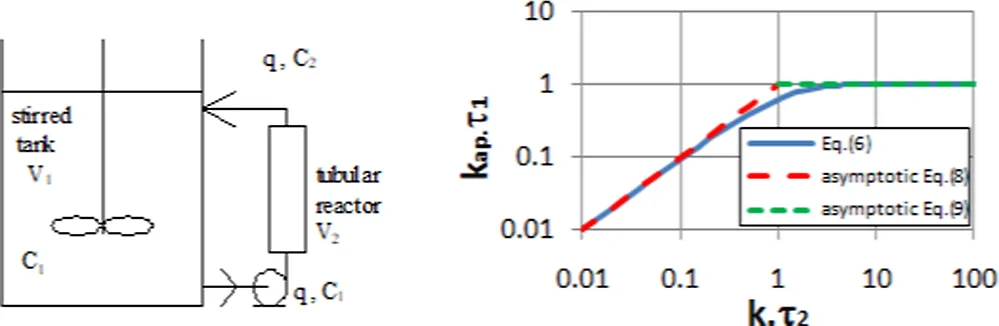

## 1 Introduction

Kinetic disguising can occur when the observed kinetics is treated using a simplified rate equation that do not account for the rigorous description of the key rate-determining steps, which may be of physical and/or chemical nature.^1^

The falsification (disguising) of kinetic parameters can occur in various situations, particularly when the analysis of experimental data relies on assumptions that are not strictly valid under the actual experimental conditions. The resulting model, being incomplete or oversimplified, transfers any errors from these invalid assumptions to the values of the effective (or apparent) kinetic parameters obtained. Consequently, the kinetic parameters thus determined—referred to as apparent or effective parameters—will be falsified.

Understanding “disguised” kinetics is important for several reasons.^1^ Reliable rate equations and accurate kinetic parameters are essential for reactor design. Furthermore, developing robust kinetic models requires first demonstrating the ability to measure fundamental rate constants for both kinetic and transport steps.^1^

In many experimental studies of photocatalyzed reactions, concentration versus time data plotted on a semilog scale often appear linear, leading to the claim of apparent first-order kinetics. However, when the initial concentration of the reagent is varied, the apparent rate constant obtained from the slope of this plot often depends on the initial concentration,^2^ clearly indicating that the true kinetics are not first-order and that some form of falsification has occurred.

Other well-known cases of kinetic disguise in liquid phase photocatalyzed reactions and in the photolysis of organic pollutants have been extensively discussed in the works of Ollis.^1–7^ The falsification of kinetics arises when some important phenomenon is omitted or incorrectly treated in the derived or assumed kinetic rate equation.^1^ Examples of such rate-influencing phenomena include limitations of mass transfer (either serial/external, between the fluid and the solid catalyst, or parallel/internal, within the porous structure of catalytic pellets), unaccounted axial dispersion in flow-through reactors, limitations in electron and hole transport in photocatalyzed reactions, and effects of light intensity and light penetration within the reactor volume. Additionally, the occurrence of parallel reactions such as catalyst deactivation can cause kinetic disguise.^8–11^ In particular, the effects of falsification in the kinetics of heterogeneous catalytic reactions due to internal and external mass transfer are well known and discussed in classical Chemical Reaction Engineering textbooks.^12–14^

Experimental systems used to study the kinetics of homogeneous and heterogeneous photochemical reactions can have various designs. One of the most commonly used systems^15–24^ is a lighted tubular reactor in which the fluid recirculates to a coupled, dark stirred tank—a configuration also known as a batch reactor operating in circulation mode. In this setup, the fluid in the tubular reactor is exposed to natural (solar) or artificial (lamp) radiation (e.g., by being placed on the focal axis of a parabolic mirror exposed to solar radiation or inside a box with one or more UV lamps). The fluid is pumped from an agitated standby tank through the tubular reactor and then returns to the tank. This reactor system (a combination of a tubular reactor and a recirculation tank) is commonly used to study the degradation of contaminants by photochemical processes such as UV photolysis, UV/H2O2, and photo-Fenton.^25–31^ This experimental setup is also widely used in electrochemical reactors, sonochemical reactors, hydrodynamic cavitational reactors, fixed-bed catalytic reactors with recirculation, and solar reactors for microalgae cultivation.

This configuration, with a tubular reactor coupled to a recirculation tank, is widely used because reagents can be fed and samples can be easily taken from the stirred tank, avoiding the more complicated process of sampling directly from the tubular reactor. Additionally, the tubular reactor can be constructed from glass or quartz with a small diameter, which, in the case of photochemical reactions, helps minimize or even neglect heterogeneity in the light field distribution across the tube’s cross section under appropriate conditions. The external recirculation also facilitates better temperature control when combined with an appropriate external heat exchanger, especially in cases involving intense radiation or significant heat dissipation phenomena such as ultrasound, electrochemical, or hydrodynamic cavitation in the tubular reactor. Besides photochemical reactors, this configuration is also commonly used in fixed-bed catalytic reactors with recirculation and sampling stirred tanks, as well as in other types of tubular reactors (sonochemical, cavitational, electrochemical, etc.) coupled to a recirculation tank.

However, special care must be taken when analyzing data collected on the concentration of the reagent in the tank over time. If the data are analyzed by treating the entire system as a batch reactor (as sometimes occurs), while the reaction actually takes place only in the tubular reactor (and not in the recirculation tank), then this approach will introduce errors into the analysis, resulting in falsified values for the kinetic parameters.

Previous works in the literature^32–34^ have discussed this problem of falsification of the kinetic parameters for small conversions per pass in the tubular reactor, and provided correction formulas for this regime, considering a Langmuir–Hinshelwood (LH) kinetics and their limiting cases of first- and zeroth-order reactions.

In the present work, this problem is revisited and a simple analysis is presented to demonstrate how an inadequate treatment of experimental data collected in this system can lead to errors in the kinetic parameters (falsified reaction order and reaction rate constant). The analysis here presented is intentionally simplified using asymptotic limits of the operating conditions (both low and high conversions per pass), and extends the results of the previous literature.^32–34^ The derived expressions relating the true and apparent parameters allow, under certain conditions, the recovery of the true parameters from their disguised values.

## 2 Analysis of the TANK-AND-TUBE Reactor

Let us consider a system consisting of a tubular reactor connected to a stirred tank with fluid circulating from one vessel to another, as shown in Figure 1. The simplifying assumptions adopted in the analysis are as follows:

(a) the chemical reaction occurs exclusively in the tubular reactor (volume V_2_) and not in the circulation tank (volume V_1_);
(b) the fluid is a liquid of constant physical properties (including density);
(c) the tubular reactor behaves like an ideal plug-flow reactor (piston flow);
(d) the tank is perfectly mixed, and no chemical reaction takes place in it;
(e) samples are periodically analyzed in the liquid inside the tank to determine the concentration of the reagent as a function of the experimental time (either by offline-analyzed small samples, whose withdrawal does not significantly alter the total volume of liquid in the system, or by some type of online sensor immersed in the liquid in the tank);
(f) the flow of liquid circulation (*q*) is kept constant throughout the experiment;
(g) the system is isothermal.
(h) the reaction can be represented by A → B, where *A* is the contaminant and *B* represent the products of the reaction. The true rate constant of the reaction is *k* and its true order is *n*, i.e., the reaction kinetics can be represented by (-r_A_) = kC^*n*^
(i) in addition, the volume of the tubular reactor is significantly smaller than the volume of the tank (V_2_ ≪ V_1_), so that the balance of A in the tubular reactor can be considered in pseudo-steady state (i.e., the dynamics of the tubular reactor are much faster than the dynamics of the comparatively large stirred tank). As shown in the literature,^31^ the tubular reactor works in pseudo-steady state if (kV_2_/q)/(1 + V_1_/V_2_) ≪ 1, which requires that (kV_2_/*q*)=(kτ_2_) ≪ 1 and/or (V_2_/V_1_) ≪ 1, inequalities that are very often satisfied in real cases.^31^

**Figure 1:**
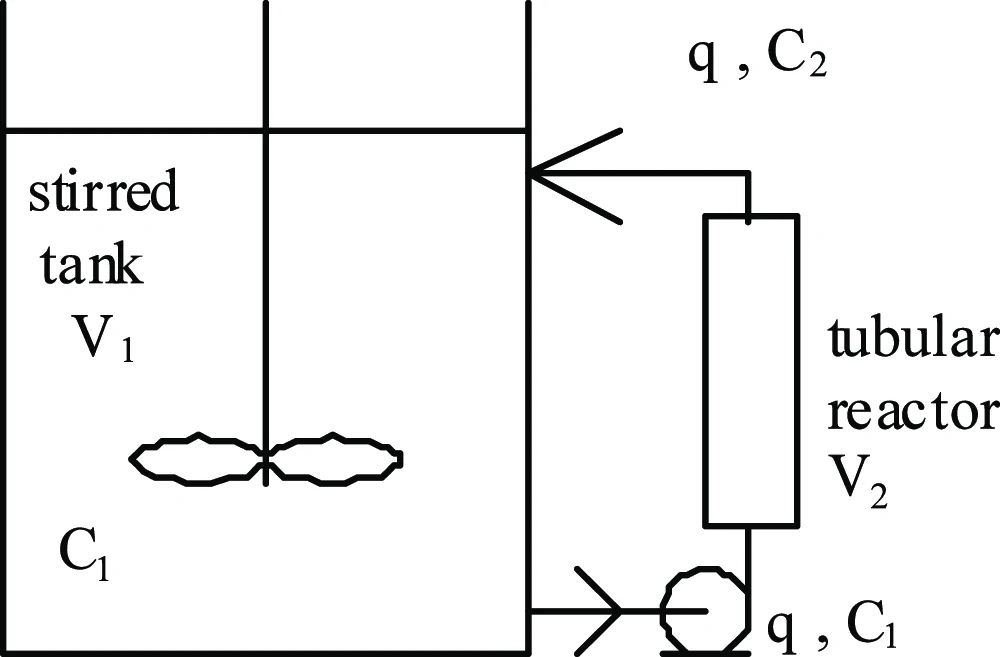
Scheme of the system under analysis, formed by a tubular reactor with the fluid circulating from and to a stirred tank, with the reaction taking place only in the tubular reactor.

With the above simplifications, the molar balance equations of A in the tank and in the tubular reactor can be written, respectively, as

1 $$V_{1}\frac{dC_{1}}{dt}=q\left(C_{2}-C_{1}\right)$$

2 $$V_{2}=-q\int_{C_{1}}^{C_{2}}\frac{dC}{kC^{n}}$$

On the other hand, if the data are treated considering the whole system (tubular reactor + stirred tank) as a batch reactor, the kinetic parameters *k* and *n* thus determined will be disguised as *k*_app_ and *n*_ap_ and the molar balance of A would be written as

3 $$\frac{dC_{1}}{dt}=-k_{ap}{\left(C_{1}\right)}^{n_{ap}}$$

Of course, because the reaction occurs only in the tubular reactor, not in the stirred tank, the analysis of the data using eq 3 is wrong and this mistake may cause significant deviations in the apparent kinetic parameters (in comparison with the true parameters), leading to falsified parameters.

The comparison between the results of eqs 1+ [Disp-formula eq2] with the results arisen from eq 3 allows one to disclose the relationships between the true (*k, n*) and the apparent (*k*_ap_, *n*_ap_) kinetic parameters. These relationships permit to recover the true parameters from the apparent (disguised) parameters, thus allowing the correction of the falsification.

## 3 Results and Discussion

### 3.1 First-Order kinetics (*n* = 1)

For the case of first-order reaction (*n* = 1), eq 2 becomes

4 $$C_{2}=C_{1}\exp\left(-kV_{2}/q\right)=C_{1}\exp\left(-k\tau_{2}\right)$$

where τ_*2*_ *= V*_2_/*q* is the space time in the tubular reactor. Substituting in eq 1, results:

5 $$\frac{dC_{1}}{dt}=-\left(\frac{1}{\tau_{1}}\right)\left[1-\exp\left(-k\tau_{2}\right)\right]C_{1}$$

where τ_*1*_ *= V*_1_/*q*. This equation was already presented in the literature^32^ (but the reader should be aware that the nomenclature used here is a little different from that adopted in ref 32). By comparing eqs 5 and 3, the following identities immediately arise:

6 $$k_{ap}=\frac{1-\exp\left(-k\tau_{2}\right)}{\tau_{1}}$$

7 $$n_{ap}=n=1$$

Equation 7 shows that the reaction order is not falsified, but eq 6 reveals that the value of the apparent rate constant is different from the value of the true rate constant. Equation 6 is valid for all values of the parameters (provided the above mentioned assumptions hold), and can be used to obtain the true rate constant k from the value of the apparent rate constant *k*_ap_. However, it is interesting to verify limiting cases by asymptotic analysis:

- if (kτ_2_) ≪ 1, i.e., low conversion per pass in the tubular reactor, then:

8 $$k_{ap}=k\tau_{2}/\tau_{1}$$

- if, otherwise, (kτ_2_) ≫ 1, i.e., high conversion per pass in the tubular reactor, then:

9 $$k_{ap}=1/\tau_{1}$$

The asymptotic eqs 8 and 9 can be easy derived from eq 6 using the well-known approximations [1-exp­(-*x*)] ≅ *x* for very low values of x, and exp­(-*x*) ≅ 0 for very high values of *x*.

Figure 2a shows the plot of (*k*_ap_τ_1_) as a function of (kτ_2_) according to eq 6. The two limiting conditions eqs 8 and 9 can be easily verified. Equation 6 can also be re-written as

10 $$\frac{k_{ap}\tau_{1}}{k\tau_{2}}=\frac{1-\exp\left(-k\tau_{2}\right)}{k\tau_{2}}$$

**Figure 2:**
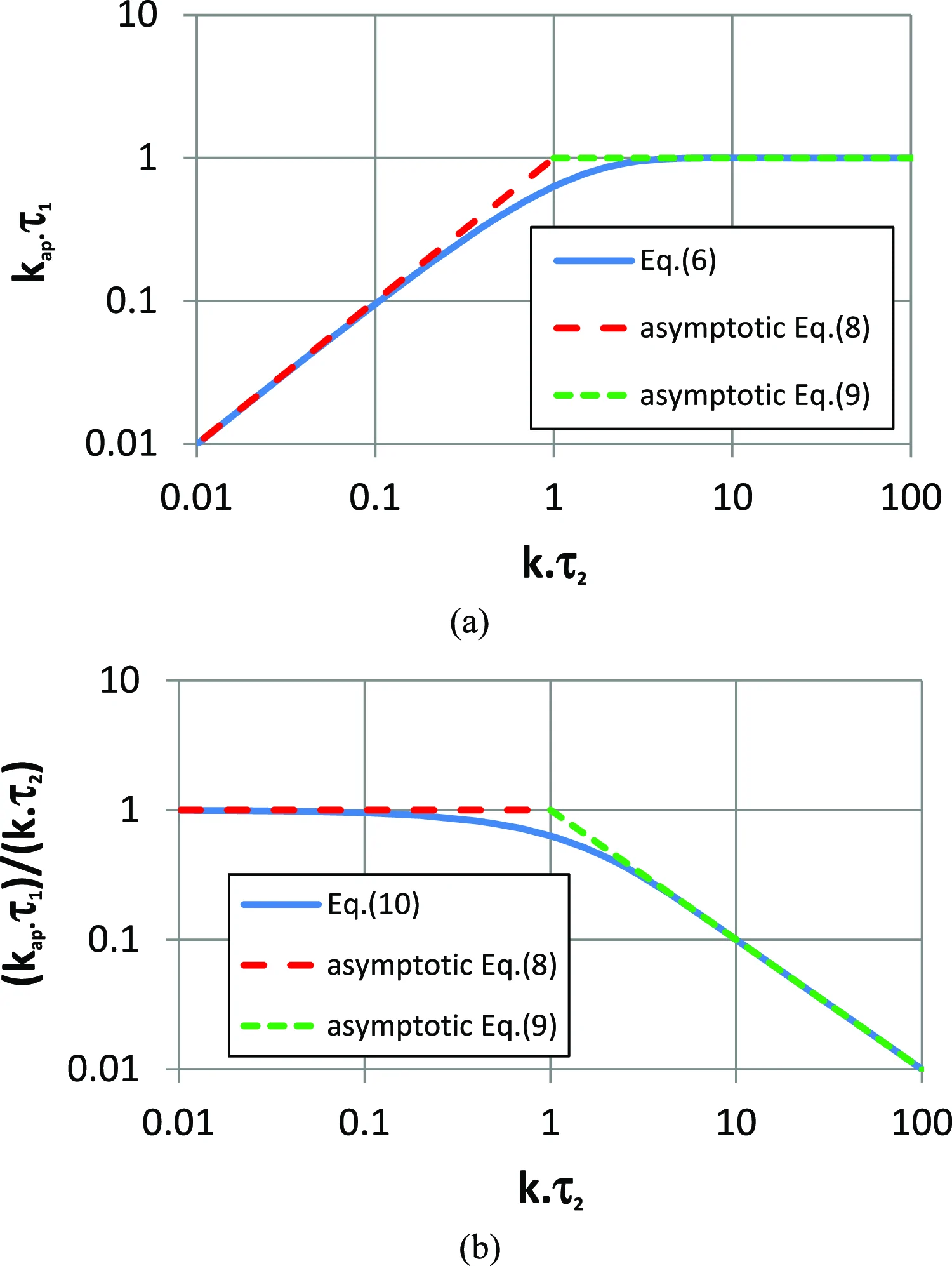
(a) Comparison between eq 6 and their asymptotic forms eqs 8 and 9; (b) Comparison between eq 10 and the asymptotic eqs 8 and 9.

As a curiosity, the plot in the form of eq 10, presented in Figure 2b, resembles pretty much the plot of the effectiveness factor of porous heterogeneous catalysts as a function of the Thiele modulus.

Figure 3 shows the percent difference between derived eq 6, which is valid for the full range of (kτ_2_) values, and their corresponding asymptotic eqs 8 and 9. This figure is useful to establish the true limit of validity of the asymptotic equations for an admitted maximum error. E.g., if one admits an error between the asymptotic equations and the derived eq 6 should be less or equal 1%, the limiting conditions (kτ_2_) ≪ 1 and (kτ_2_) ≫ 1 become, in practice, (kτ_2_) < 0.02 and (kτ_2_) > 4.62. If the maximum admitted error is 0.5%, then the limiting conditions (kτ_2_) ≪ 1 and (kτ_2_) ≫ 1 become, effectively, (kτ_2_) < 0.01 and (kτ_2_) > 5.3.

**Figure 3:**
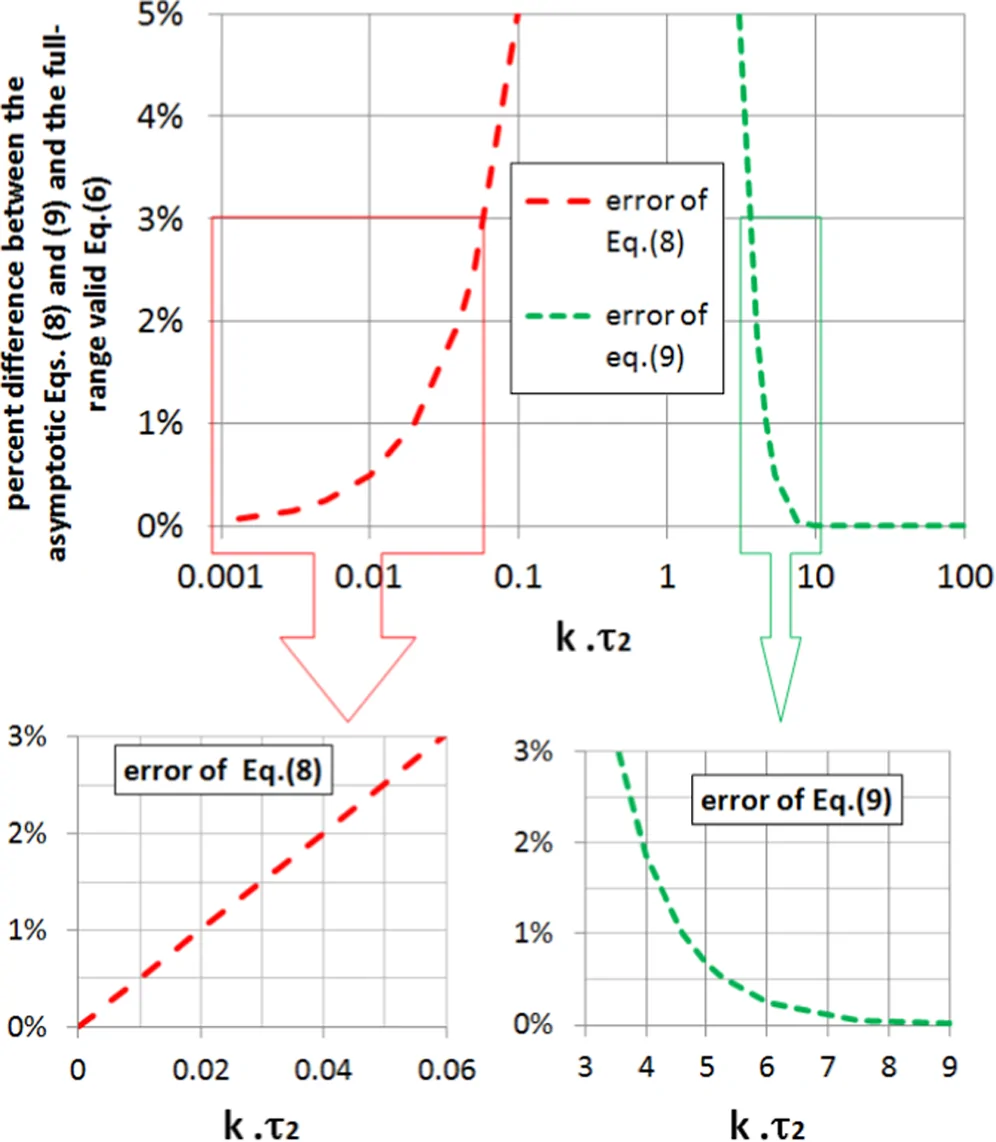
Percent error between the asymptotic eqs 8 and 9 and the full-range valid eq 6. For a given admitted percent error, this plot allows to establish the practical limits for the validity of the asymptotic equations.

### 3.2 Second-Order kinetics (*n* = 2)

For the case of second-order kinetics (*n* = 2), eqs 2 and 1 become, respectively:

11 $$C_{2}=C_{1}\left[\frac{1}{1+kC_{1}\tau_{2}}\right]$$

12 $$\frac{dC_{1}}{dt}=-k_{ap}{\left(C_{1}\right)}^{n_{ap}}=-\left(\frac{1}{\tau_{1}}\right)\left[\frac{kC_{1}\tau_{2}}{1+kC_{1}\tau_{2}}\right]C_{1}$$

For this case, it is not possible to derive a simple analytical expression for *k*_ap_ and n_ap_ (analogous to eqs 6 and 7), but it is still possible to obtain the asymptotic limiting cases as:

- for low conversion per pass in the tubular reactor, (kC_1_τ_2_) ≪ 1, results:

13 $$k_{ap}=\frac{k\tau_{2}}{\tau_{1}}$$

14 $$n_{ap}=n=2$$

- for high conversion per pass in the tubular reactor, (kC_1_τ_2_) ≫ 1, results:

15 $$k_{ap}=\frac{1}{\tau_{1}}$$

16 $$n_{ap}=1$$

### 3.3 Zeroth-Order Kinetics (*n* = 0)

For the case of zeroth-order kinetics (*n* = 0), eqs 2 and 1 can be written, respectively, as:

17 $$C_{2}=\{\begin{array}{ll} C_{1}-k\tau_{2} & \text{if},C_{1}>k\tau_{2}\\ 0 & \text{if},C_{1}\leq k\tau_{2} \end{array}$$

18 $$\frac{dC_{1}}{dt}=-k_{ap}{\left(C_{1}\right)}^{n_{ap}}=\{\begin{array}{ll} -\left(\frac{k\tau_{2}}{\tau_{1}}\right) & \text{if},C_{1}>k\tau_{2}\\ -\left(\frac{C_{1}}{\tau_{1}}\right) & \text{if},C_{1}\leq k\tau_{2} \end{array}$$

thus resulting in the following exact results:

- if (k τ_2_)<C_1_ then:

19 $$k_{ap}=\frac{k\tau_{2}}{\tau_{1}}$$

20 $$n_{ap}=n=0$$

- if (k τ_2_) ≥ *C*_1_ and *C*_2_ = 0, then:

21 $$k_{ap}=\frac{1}{\tau_{1}}$$

22 $$n_{ap}=1$$

Note that, for particular case of zeroth-order kinetics, the asymptotic cases are exactly the same as the exact results given in eqs 19–22.

### 3.4 Generic n*th*-Order kinetics (*n* ≠ 1)

For the case of *n*th-order kinetics (n≠1), eqs 2 and 1 can be written, respectively, as:

$$C_{2}=C_{1}{\left[1+\left(n-1\right)kC_{1}^{n-1}\tau_{2}\right]}^{1/(1-n)}$$ 23

$$\frac{dC_{1}}{dt}=-k_{ap}{\left(C_{1}\right)}^{n_{ap}}=\left(\frac{C_{1}}{\tau_{1}}\right)\left\{{\left[1+\left(n-1\right)kC_{1}^{n-1}\tau_{2}\right]}^{1/(1-n)}-1\right\}$$ 24

For this case, it is not possible to obtain a simple analytical expression for *k*_ap_ and n_ap_ valid for the entire range of values. The asymptotic limiting cases are:

- for low conversion per pass in the tubular reactor, (kC_1_^n–1^τ_2_) ≪ 1, results:

25 $$\frac{dC_{1}}{dt}=-k_{ap}{\left(C_{1}\right)}^{n_{ap}}=\left(\frac{C_{1}}{\tau_{1}}\right)\left\{-kC_{1}^{n-1}\tau_{2}\right\}=-\left(\frac{k\tau_{2}}{\tau_{1}}\right)C_{1}^{n}$$

26 $$k_{ap}=\frac{k\tau_{2}}{\tau_{1}}$$

27 $$n_{ap}=n$$

- for high conversion per pass in the tubular reactor, (kC_1_^*n*–1^τ_2_) ≫ 1, results:

28 $$\frac{dC_{1}}{dt}=-k_{ap}{\left(C_{1}\right)}^{n_{ap}}=\left(\frac{C_{1}}{\tau_{1}}\right)\left\{-1\right\}=-\left(\frac{1}{\tau_{1}}\right)C_{1}$$

29 $$k_{ap}=\frac{1}{\tau_{1}}$$

30 $$n_{ap}=1$$

Equations 25 and 28 can be derived from eq 24 using the asymptotic approximations [1+(*n*-1)*x*]^1/(1‑*n*)^ ≅ (1-*x*) for very low values of x, and [1+(*n*-1)*x*]^1/(1‑*n*)^ ≅ 0 for very high values of *x*.

### 3.5 Illustrative Calculation

In this section we presented some illustrative calculations to test and show the usefulness of the results. D’Avila^35^ studied the degradation of herbicide amicarbazone (AMZ) by the TiO2/UV process in a recirculation reactor equipped with 10 tubes of borosilicate glass (internal diameter 2.92 cm, length 140 cm, irradiated volume 1 L per tube) placed in the focal point of parabolic collectors irradiated by solar light. In all experiments, the recirculation flow rate was set to 500 L/h and the total volume of solution was 20 L (volume of liquid in the tank V_1_ = 10 L, τ_1_ = 1.2 min). Dissolved oxygen, pH, temperature and incident solar irradiation were measured during the experiments. Effects of TiO2 concentration, initial AMZ concentration and number of tubes exposed to the solar irradiation were studied. We selected experiments carried out under similar conditions (TiO_2_ concentration, initial AMZ concentration, intensity of the solar irradiation, etc.), where only the irradiated volume (V_2_) was changed.

Figure 4a shows the measurements of [AMZ] versus time for the Experiment 3 (7 irradiated tubes, V_2_ = 7 L, τ_2_ = 0.84 min) and Experiment 11 (3 irradiated tubes, V_2_ = 3 L, τ_2_ = 0.36 min). The data are reasonably fitted by the first-order kinetics and the apparent rate constants were *k*_ap_ = 0.0150 min^–1^ for Exp. 3 and *k*_ap_ = 0.0072 min^–1^ for Exp. 11.

**Figure 4:**
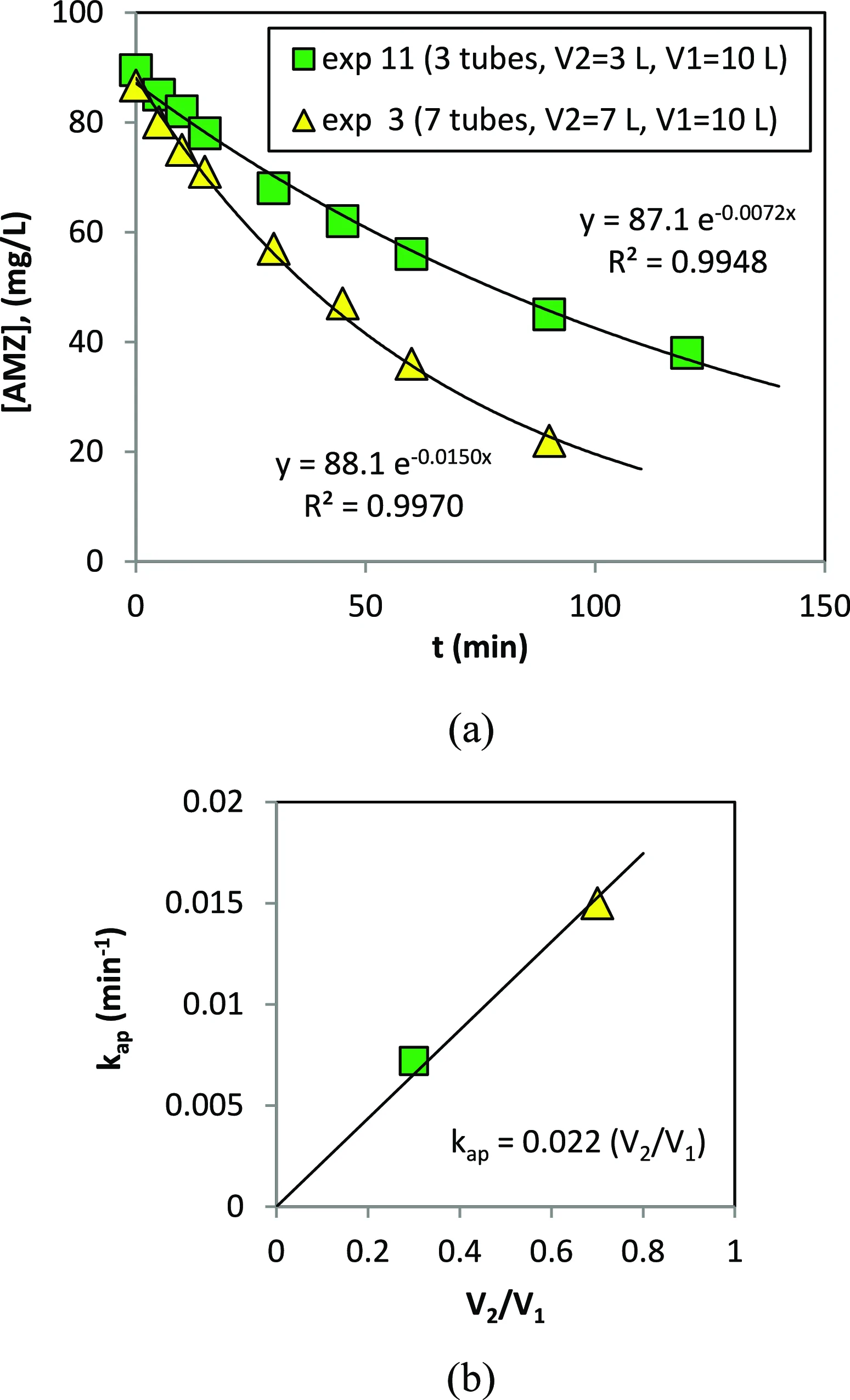
(a) Concentration history measured in experiments 3 and 11 of D’Avila.^35^ (b) Test of asymptotic eq 8 to determine the true rate constant.

Applying eq 6 to recover the value of the true rate constant:

31 $$k=\frac{-\ln\left(1-k_{ap}\tau_{1}\right)}{\tau_{2}}$$

we obtain *k* = 0.0216 min^–1^ for Exp. 3 and *k* = 0.0241 min^–1^ for Exp. 11. The correction (difference between the apparent and true rate constants) is significant.

An alternative way to find the values of the true rate constant is through the asymptotic eq 8, resulting in *k* = 0.0214 min^–1^ for Exp. 3 and *k* = 0.0241 min^–1^ for Exp. 11, values quite close to those obtained from eq 31. This confirms that the asymptotic equation is indeed valid for this case (where *k*.τ_2_ (≈kap.τ_1_) ≪ 1).

Figure 4b presents the plot of *k*_ap_ versus the ratio (V_2_/V_1_) = (τ_2_/τ_1_), which serves as a test of asymptotic eq 8, showing that the two points are well aligned to a straight line passing through the origin. According to eq 8, the true rate constant can be determined from the slope of this line, k = 0.022 min^–1^.

Klausner et al.^34^ presented an example for a different reaction (photocatalytic degradation 2-chlorophenol) for two different recirculation flow rates. They presented a plot similar to Figure 4b, but using the ratio (V_2_/(V_1_+V_2_)) instead of (V_2_/V_1_) in the horizontal axis. Their volumes of tubular reactor and tank are such that the ratios (V_2_/(V_1_+V_2_)) < 0.056 (which correspond to (V_2_/V_1_) < 0.053). It is interesting to note that, in the example here presented, the volume ratios (V_2_/V_1_) were considerably higher (0.3 and 0.7), but the results of the analysis are still consistent.

## 4 Summary of the Results and Concluding Remarks

Table 1 summarizes the results found from the asymptotic analysis made for zeroth- first-, second- and *n*th-order reactions. It is interesting to note that the results for the asymptotic conditions are exactly the same for the different true reaction orders studied and can summarized as.

- for low conversion per pass in the tubular reactor (*kC*_1_^*n*–1^τ_2_) ≪ 1, the reaction order is not falsified (*n*_ap_ *= n*) but the rate constant is falsified to *k*_ap_ = *k*τ_2_/τ_1_
- for high conversion per pass in the tubular reactor (*kC*_1_^*n*–1^τ_2_) ≫ 1, the reaction order is shifted to an apparent first-order (*n*_ap_ = 1) and the rate constant is falsified to *k*_ap_ = 1/τ_1_

**Table 1:** Falsification of the Kinetic Parameters in Tubular Reactor With Recirculation to a Stirred Tank (Limiting Cases)

	asymptotic condition: low conversion per pass			asymptotic condition: high conversion per pass		
true reaction order *n*	condition	*n*_ap_	*k*_ap_	condition	*n*_ap_	*k*_ap_
0	(k τ_2_) < C_1_ ^a^	0	$k_{ap}=\frac{k\tau_{2}}{\tau_{1}}$	(k τ_2_) ≥ C_1_ ^a^	1	$k_{ap}=\frac{1}{\tau_{1}}$
1	(k τ_2_) ≪ 1	1	$k_{ap}=\frac{k\tau_{2}}{\tau_{1}}$	(k τ_2_) ≫ 1	1	$k_{ap}=\frac{1}{\tau_{1}}$
2	(k C_1_τ_2_) ≪ 1	2	$k_{ap}=\frac{k\tau_{2}}{\tau_{1}}$	(k C_1_τ_2_) ≫ 1	1	$k_{ap}=\frac{1}{\tau_{1}}$
N	(k C_1_^*n*–1^τ_2_) ≪ 1	*n*	$k_{ap}=\frac{k\tau_{2}}{\tau_{1}}$	(k C_1_^n–1^τ_2_) ≫ 1	1	$k_{ap}=\frac{1}{\tau_{1}}$

a For a zeroth-order reaction, according to eq 18, it is enough to use < instead of ≪ in these inequalities.

This analysis demonstrates that data collected from a system consisting of a tubular reactor and a recirculation stirred tank must be carefully analyzed; treating the entire system as a batch reactor will result in falsification of the kinetic parameters.

For low conversion per pass in the tubular reactor (k C_1_^*n*–1^τ_2_) ≪ 1, the reaction order *n* is not falsified, but the apparent rate constant is disguised to (k τ_2_/τ_1_). In this case, one could obtain the true rate constant by inverting eq 8 as:

32 $$k=\frac{k_{ap}\tau_{1}}{\tau_{2}}$$

Notably, even for a slow reaction, the rate constant is still falsified, although the reaction order remains unaffected.

On the other hand, for high conversion per pass in the tubular reactor, i.e., (k C_1_^*n*–1^τ_2_) ≫ 1, the apparent order is disguised as 1 (i.e., apparent first-order) regardless of the true order n, and the apparent rate constant becomes (1/τ_1_), regardless of the true rate constant *k*. This shift to apparent first-order behavior can be interpreted as the system dynamics being governed by the balance of A in the stirred tank (eq 1), which inherently follows first-order dynamics due to dilution. Furthermore, in this case, it is not possible to recover the true rate constant *k* from the value of the obtained apparent rate constant *k*_ap_ because for high conversion per pass, C_2_≅ 0, thus no information about the reaction kinetics is transferred to the tank, where concentrations are monitored. This is also illustrated in Figure 2a: for *k*_ap_τ_1_ = 1, the green line indicates that there are infinite values of k τ_2_, making it impossible to determine *k* from *k*_*ap*_. Under this condition, the reaction kinetics in the tubular reactor are too fast, so the changes in the concentration in the tank (and the overall process) are fully governed by the first-order dilution process in the tank. As a result, the experimental data of *C*_1_(*t*) will fit perfectly to a straight line in a semilog plot (indicating an overall apparent first-order behavior), even when the true reaction kinetics is not first-order.

Some works in the literature^26–30^ expressed the mass balance of A in a system formed by a tubular reactor coupled to a recirculation tank (*V*_tot_ = V_1_+V_2_) in the form:

33 $$\frac{dC_{1}}{dt}=\frac{\left(-r_{A}\right)V_{2}}{V_{tot}}=\frac{\left(-r_{A}\right)V_{2}}{V_{1}+V_{2}}=\frac{\left(-r_{A}\right)\tau_{2}}{\left(\tau_{1}+\tau_{2}\right)}$$

which is equivalent to the derived eqs 5, 12, 18, and 24 when τ_2_≪τ_1_. This means that eq 24 is able to correctly account for the disguise of the parameters for the cases of low conversion per pass.^32–34^ However, this is not true for the other asymptotic case (high conversion per pass).

Wolfrum and Turchi^33^ presents an elegant analysis accounting for that the outlet concentration of the tubular reactor at a given instant *C*_2_(*t*) is related to the inlet concentration one residence time ago C_1_(t-τ_2_), thus accounting for the transient regime for the PFR. Their analysis justifies the use of ratio V_2_/(V_1_+V_2_) or τ_2_/(τ_1_+τ_2_) as in eq 33, instead of the ratio (V_2_/V_1_) or (τ_2_/τ_1_) of our eqs 8, 13, 19 and 26; however, their derivation also assumed (so it is limited to) small values of (τ_2_), condition under which these two ratios are equivalent V_2_/(V_1_ + V_2_) ≈ (V_2_/V_1_).

The results here presented are valid and are consistent with those previously presented in the literature.^32–34^
